# The Role of Additional K-Wires on AO Type C Distal Radius Fracture Treatment with External Fixator in Young Population

**DOI:** 10.1155/2019/8273018

**Published:** 2019-01-01

**Authors:** Ivan Micic, Erica Kholinne, Yucheng Sun, Jae-Man Kwak, In-Ho Jeon

**Affiliations:** ^1^Clinic for Orthopaedic Surgery and Traumatology, Clinical Center Nis, Nis, Serbia; ^2^Department of Orthopedic Surgery, St. Carolus Hospital, Jakarta, Indonesia; ^3^Department of Orthopedic Surgery, Asan Medical Center, University of Ulsan College of Medicine, Seoul, Republic of Korea; ^4^Department of Hand Surgery, Affiliated Hospital of Nantong University, Nantong, Nantong University, Jiangsu, China

## Abstract

**Objectives:**

Several methods have been proposed to treat AO type C distal radius fracture. External fixator has gained popularity for its simple procedure and rapid recovery. Some surgeons suggested that additional K-wires may play a critical role in the outcome. The purpose of study is to evaluate the role of additional K wires in treating distal radial fracture with external fixator regarding its outcome.

**Material and Methods:**

From January 2006 to January 2010, 40 patients with AO type C distal radius fracture were treated with external fixator, with (EF) or without additional K wires (EFK). Radiologic outcome parameters include radial inclination, volar tilt, radial length, and the presence of radiocarpal arthritis according to Knirk and Jupiter. Clinical outcomes include New York Orthopedic Hospital (NYOH) wrist scoring scale.

**Results:**

Radiographic outcome showed significant difference in regard of articular congruency at the final follow-up with the EFK group showing the advantage in maintaining the articular incongruity. NYOH wrist scoring scale showed no significant difference between both groups at final follow-up. The amount of articular step-off was less in EFK group with significant statistical finding on the final follow up.

**Conclusion:**

Both EF and EFK technique were able to provide satisfactory result in treating AO type C distal radius fractures. We observed that EFK is superior in reducing the number of radiocarpal arthritic changes compared to EF group due to its superiority in reducing articular step-off.

## 1. Background

The extra-articular distal radius fractures occur frequently in osteoporotic geriatric group, while the intra-articular type is more frequent in young adult patients with high-energy trauma [1]. The high energy injury pathomechanism involved axial load transfer from hand to the articular surface of distal radius. This causes shearing force which leads to impacted fracture and marked displacement [2]. Most of this high energy fracture is unstable and classified as AO type C fracture. Several studies have reported the comparison between external and internal fixation and concluded that volar locking plate (VLP) is superior to the external fixator (EF) [1, 3–5]. Nevertheless, EF showed its distinctive advantages with nondemanding surgical procedures and unnecessity for hardware removal surgery for intra-articular distal radius fracture [3, 6, 7].

External fixation for distal radius fracture is applied after ligamentotaxis achieved following fracture reduction [8]. Unfortunately, comminuted fracture type is often difficult to reduce and maintain. Hence, additional K-wires are needed as a reduction tool and later provide additional stability to fracture site. To date, not many studies focus on the role of additional K-wires in external fixator. Therefore, the aim of our study is to define whether additional K-wires in external fixator will influence the outcome of distal radius fracture in young population.

## 2. Materials and Method

**Figure 1:**
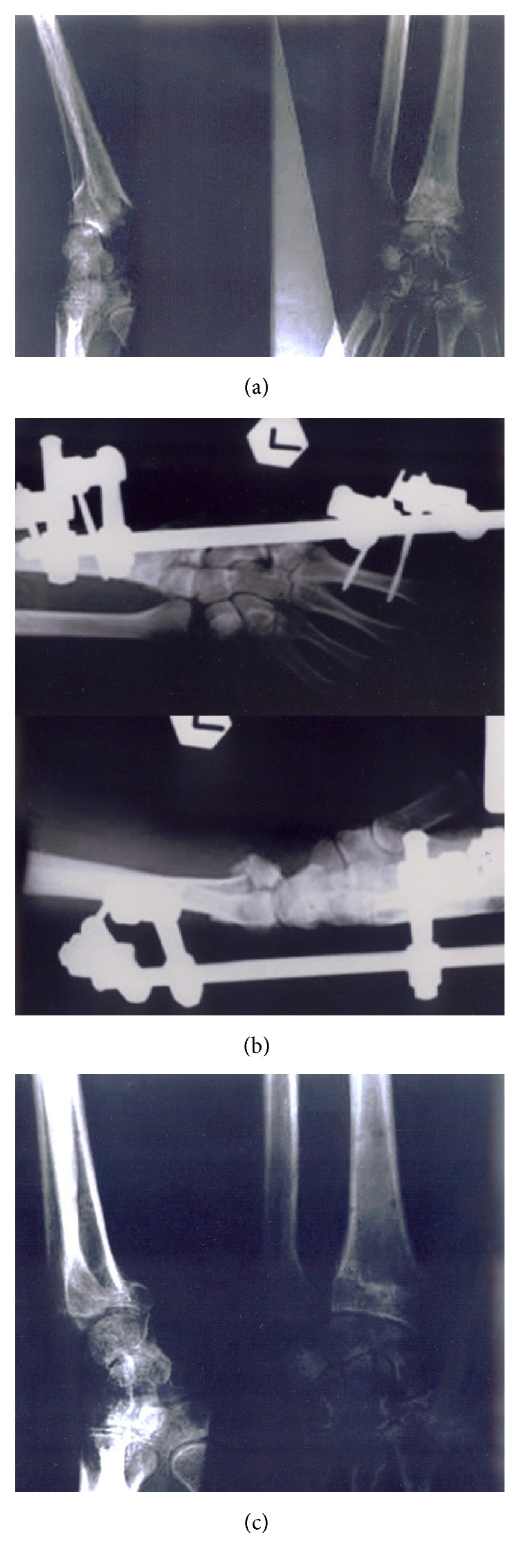
Preoperative (a), postoperative (b), and final follow-up radiographs of EF group (c).

**Figure 2:**
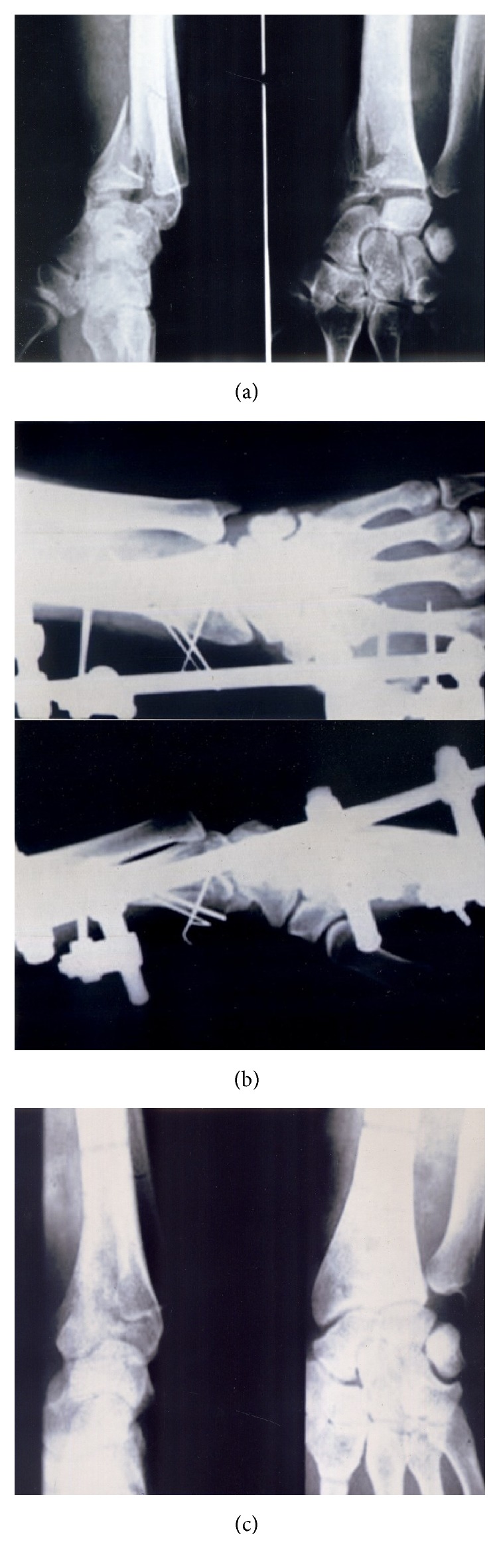
Preoperative (a), postoperative (b), and final follow-up radiographs of EFK group (c).

This retrospective cohort study enrolled forty patients from January 2006 to 2010. Institutional review board approval was obtained prior to study. The power of study was estimated at 0.8 with 10% of type 1 error and the above sample size. The inclusion criteria were patients aged between 18 and 50 years, AO type C distal radius fractures, dorsal comminution, > 10 degrees' dorsal tilt, > 3mm radial shortening, and >1 mm intra-articular step-off after manipulative reduction. Patients with medical contraindications, open fractures, bilateral fractures, concomitant injuries, previous wrist injuries and diseases, and AO type A/B fractures were excluded. Preoperative radiographic assessment was obtained for all patients. All patients were treated with closed reduction under fluoroscopic guide and followed by external fixation (EF group) (Figures 1(a) and 1(b)) or with additional K-wires fixation (EFK group) (Figures 2(a) and 2(b)).

### 2.1. Surgical Technique

All patients were treated with closed manipulation and splinting prior to surgical intervention. Patients whose radiographs following closed manipulation demonstrated unacceptable reduction of the metaphyseal-diaphyseal junction were scheduled for surgical intervention. Procedure was performed under general anesthesia with patient in supine position on radiolucent table. A 250mmHg tourniquet was applied proximally. Stab incisions were made on the dorsal-radial side superior from the extensor pollicis longus tendon and on the lateral side of the second metacarpal. Two 4-mm pins were inserted proximally and two were inserted distally in a different trajectory angle. Pins were then connected to the bar with clamps. Traction was applied, respectively, under fluoroscopic control after satisfactory radiologic parameters (radial inclination, volar tilt, and radial length) were achieved.

In EFK group, the articular fragment was reduced percutaneously using pointed reduction forceps followed by percutaneous 0.45-in K-wire insertion under fluoroscopy guidance through either the radial styloid and/or the intermediate column of the wrist. External fixator was applied accordingly. All patients were treated by single orthopedic surgeon.

### 2.2. Postoperative Management

Active and passive range-of-motion exercise of the digits and the elbow were initiated on the second day after operation under the supervision of a hand physical therapist. Wound dressing was carried out once daily. The K-wires were removed at six weeks after operation and two weeks later external fixators were removed subsequently. Range-of-motion exercise of the wrist and hand exercises was encouraged afterwards.

### 2.3. Evaluation

Patients were followed up for at least 1 year with the interval at 3, 6, and 12 months. The mean of final follow-up is 33 months (range: 26 to 55 months). Demographic data included age, sex, hand dominance, and fracture type. Serial postoperative outcome follow-up assessment was done by orthopedic surgeon with NYOH wrist scoring scale. Postoperative radiographic evaluation was carried out by radiologist for radial inclination, volar tilt, radial length, articular congruency status, amount of articular step-off, and the presence of radiocarpal arthritis according to Knirk and Jupiter (Figures 1(c) and 2(c)). Pre- and postoperative radiographic evaluations were compared.

### 2.4. Statistical Analysis

The* t*-test was used to compare the ages, sex, hand dominance, fracture type, NYOH wrist scoring scale (pain, function, movement, grip strength, and presence of arthrosis), and radiographic parameters (radioulnar angle, volar angle, radial length, and articular congruency). Chi-square test was used to compare the articular step-off in both EF and EFK group. The relationship between articular step-off to the presence of radiocarpal arthritis, mobility, and pain was analyzed with Pearson correlation test. A* p* value ≤ 0.05 was considered to be statistically significant.

## 3. Results

The mechanism of injury was fall on an outstretched hand for twenty-four patients and motor-vehicle accident for sixteen patients. Patients' demographics are shown in Table 1. All fractures healed accordingly without any additional intervention. No distal radioulnar instability was found at the final follow-up. Superficial pins-track infection developed in four (10%) patients. Oral antibiotic and daily dressing care around the pins was performed accordingly. Deep bone infection was not observed at final follow-up. There was no refracture or tendon rupture observed. No patients reported digital stiffness or reflex sympathetic dystrophy at the end of follow-up.

We found a significant difference of patients' demographics in terms of sex ratio between two groups (*p*= 0.048). Functional outcome according to NYOH wrist scoring scale showed no significant difference between both groups at final follow-up (Table 1). Radiographic outcome showed significant difference in regard of articular congruency at the final follow-up with the EFK group showing the advantage in maintaining the articular incongruity (*P*<0.001) (Table 2). The amount of articular step-off was less in EFK group with significant statistical finding at the final follow-up (Table 3).

The relationship between articular step-off and the presence of radiocarpal arthritis showed a significant negative linear correlation for EFK group (p < 0.001). Articular step-off also showed a negative linear correlation with pain but nonstatistically significant. The relationship between articular step-off and mobility showed a positive linear correlation with no statistical significance for both groups (Table 4).

At the final follow-up, 6 patients in EF group and 4 patients in EFK group presented with articular incongruence with radiocarpal arthritic changes in their radiographic examination. Only one patient in EFK group showed radiocarpal arthritic changes despite congruent articular surface of the wrist. Overall, the total incidence of arthritis in EF and EFK group is 30% and 20%, respectively.

## 4. Discussion

AO type C distal radius fractures are mostly hard to reduce and stabilize due to their multifragmentary and unstable characteristics. The aim of treatment is to achieve congruent articular surface and correct axial malalignment while maintaining good reduction to preserve function. Inability to achieve congruent articular surface has been shown to cause posttraumatic arthritic changes in the wrist joint.[9]

Many strategies have been widely studied in terms of distal radius fracture fixation type. VLP has gained its popularity for the treatment of distal radius comminuted fracture. Direct visualization allows us to achieve stable rigid fixation with direct manipulation to the fracture fragments. Thus, early mobilization will result in good wrist function. Studies had been done to compare external fixation with percutaneous pin and open reduction with plate fixation for distal radius fracture [1, 3–5]. Wright et al. and Rizzo et al. reported better recovery in open reduction and internal fixation (ORIF) compared with EF group in ulnar variance and volar tilt [10, 11]. Williksen et al. and Roh et al. found that ORIF group showed advantage in ulnar variance compared with EF group [4, 12]. Kreder et al. and Jeudy et al. found that articular step-off status in ORIF group is superiorly maintained [13, 14]. Most of the results are in favor of volar locking plate, for its radiological and clinical outcome [1, 3, 4].

To date, there is still no cogent conclusion to favor VLP fixation over external fixation or vice versa. External fixation relies solely on ligamentotaxis to correct and maintain fracture alignment until healing is achieved [15]. Being less invasive and hence with less surgical trauma and moreover low technically demanding tools to apply, external fixator is favorable for some surgeons. Lin et al. reported that external fixation is widely used to treat these fractures for its minimally invasive technique [16]. External fixation application did not cause any trauma to soft tissue adjacent to fracture site and thus prevented devascularization of the fracture site [17, 18]. The unnecessity for further implant removal surgery is considered to be a favorable factor in some developing countries. Therefore, health care cost would be reduced. EF is also able to prevent further complications that could arise from secondary surgery (implant removal) since EF removal is feasible to be done in office setting under local anesthesia manner [3]. In a multicenter randomized control trial, Kreder et al. noted that indirect reduction and percutaneous fixation were associated with a rapid recovery and better functional outcome in comparison with open reduction and internal fixation [14]. In a prospective randomized control trial, Williksen et al. found that EF surgery was simpler and needed less time compared with VLP surgery, though their result was still in favor of the VLP fixation [5].

Our study showed homogenous demographic data for age, hand dominance, and fracture type in our sample. Male predominance was seen in EFK group due to the possibility of having higher trauma energy pathomechanism. NYOH wrist scoring scale showed better outcome in EFK group especially in clinical parameters (pain, function, ROM, and grip strength) without statistical significance. Radiological parameter (arthrosis) only showed slightly higher score in EFK group without statistical significance. The mainstay of having an additional K wire in EF is to maintain articular surface reduction and provide additional stability. Hence, we postulated that this could reduce and minimize the incidence of secondary radiocarpal arthritis. All radiographic parameters were corrected at immediate postoperative radiographic evaluation. Our study showed that radial inclination, volar tilt, and radial length correction were not statistically significant for both groups in immediate postoperative and final follow-up time frame. We found that only articular step-off parameter showed significant correction in the final follow-up in both groups. Moreover, our study showed that there is strong statistical correlation for the presence of radiocarpal arthritic changes in regard of coexistent articular step-off. EF was able to maintain all radiographic parameters except for articular congruency. In our study, the additional K-wires were removed at six weeks postoperatively. At this time fracture has consolidated and allowed radiological parameters to be maintained. This explains why EFK group have better articular congruence because the additional K-wires maintain articular reduction until consolidation is achieved.

According to Knirk and Jupiter scale [19], there were 4 patients in EFK group and 6 patients in EF group who showed radiocarpal arthritis. All patients in EFK groups presented with minimal arthritic changes while 6 patients in EF group presented with 4 minimal, 1 mild, and 1 severe arthritic change. The arthritis incidence of our study is 33.3%, which is low compared to study by Knirk and Jupiter with 65% prevalence with a mean of 6.7 years' follow-up [19]. Another study by Arora et al. reported that 44% of elderly patients treated with VLP fixation presented with radiocarpal arthritis and 62% of them who received conservative treatment also had arthritis changes in the end [20]. Forward and colleagues reported that 68% of patients with intra-articular fractures developed radiographic evidence of arthritis at a mean follow-up of 38 years [7]. Our short-term follow-up explained the reason of having lower number of arthritic changes. Some authors suggested that patients should be counseled on the long-term risk of developing radiographic arthritic changes following distal radius fixation. On the other hand, the age of patients in our study is relatively young (range from 18 to 50 years); hence the incidence of arthritis is lower. The positive correlation between patient's age and arthritic changes has been studied well [21].

Our study has several limitations. The current study was a retrospective, nonrandomized, comparative trial with small sample size and short follow-up term with a single outcome assessment tool (NYOH wrist scoring scale). We suggested that a future randomized clinical trial will provide cogent conclusion towards this issue.

## 5. Conclusion

Both EF and EFK techniques were able to provide satisfactory result in treating AO type C distal radius fractures. We observed that EFK is superior in reducing the number of radiocarpal arthritic changes compared to EF group due to its superiority in reducing articular step-off.

## Figures and Tables

**Table 1:** Patients' demographics and NYOH wrist scoring scale assessment.

***Variable***	*EF*	*EFK*	*P-value*
*Age (years)*	41(6)	39(10)	*0.504*
*Range*	27-49	18-48	
*Sex*			*0.048∗*
*Male*	9	16	
*Female*	11	4	
*Hand dominance affected*			*0.191*
*Yes*	15	10	
*No*	5	10	
*AO type of fracture*			*0.939*
*C 1.*	4	4	
*C 2.*	7	8	
*C 3.*	9	8	
*Pain(points)*	17.8 (2.4)	18.6 (2.0)	*0.258*
*Function(points)*	26.8 (3.4)	27.8 (3.0)	*0.328*
*Movement(points)*	12.9 (1.7)	13.3 (1.8)	*0.430*
*Grip(points)*	12.7 (2.1)	12.9 (1.9)	*0.752*
*Arthrosis*			*0.545*
*No*	14	16	
*Minimal*	4	4	
*Moderate*	1	0	
*Severe*	1	0	
*NYOH Total points*	86.7 (11.8)	91.3 (10.0)	*0.195*
*Results*			*0.806*
*Excellent*	10	12	
*Fair*	1	1	
*Good*	9	7	

EF: external fixation group; EFK: external fixation adjuvant K-wires group. NYOH: New York Orthopaedic Hospital wrist scoring scale consists of pain, function, movement, and grip.

*∗*Statistically significant (p<0.05).

**Table 2:** Radiographic assessment.

		EF	EFK	P value
Radial inclination (degree)	Preoperation	2,9 (1,1)	4,2 (1,2)	0,415
	Postoperation	20,4 (0,4)	20,1 (0,3)	0,574
	Final follow-up	19,5 (0,4)	19,3 (0,5)	0,775

Volar tilt (degree)	Preoperation	-6,1 (1,8)	-6,7 (1,2)	0,355
	Postoperation	9,6 (0,3)	9,2 (0,3)	0,289
	Final follow-up	7,7 (0,5)	8,2 (0,7)	0,201

Radial length (mm)	Preoperation	-0,4 (1,2)	1,0 (0,8)	0,513
	Postoperation	11,2 (0,3)	10,4 (0,4)	0,123
	Final follow-up	10,7 (0,4)	10,1 (0,4)	0,278

Articular incongruity (mm)	Preoperation	2,5 (0,3)	2,5 (0,3)	0,910
	Postoperation	0,4 (0,1)	0,1 (0,1)	0,103
	Final follow-up	1,0 (0,2)	0,1 (0,1)	≤0.001*∗*

*∗*Statistically significant (p<0.05).

**Table 3:** Articular step-off assessment.

		EF	EFK	P value
Articular step-off ≥ 2mm	Preoperation	15	13	0.490
	Postoperation	2	0	0.487
	Final follow-up	6	0	0.020*∗*

*∗*Statistically significant (p<0.05).

**Table 4:** Correlation of articular step-off to radiocarpal arthritic changes, mobility, and pain.

Pearson correlation test	EF	EFK
Articular step-off ≥ 2mm – radiocarpal arthritic changes	r = (-) 0.306 p = 0.189	r = (-) 0.793 p ≤ 0.001*∗*

Articular step-off ≥ 2mm – mobility	r = (+) 0.120 p = 0.614	r = (+) 0.111 p = 0.641

Articular step-off ≥ 2mm – pain	r = (-) 0.112 p = 0.638	r = (-) 0.250 p = 0.288

*∗*Statistically significant (p<0.05).

## Data Availability

The data used to support the findings of this study are available from the corresponding author upon request.
